# Adding Structured Components to Home Visitation to Reduce Mothers’ Risk for Child Maltreatment: a Randomized Controlled Trial

**DOI:** 10.1007/s10896-023-00509-7

**Published:** 2023-02-13

**Authors:** Trudy van der Stouwe, Patty Leijten, Jessica J. Asscher, Maja Deković, Claudia E. van der Put

**Affiliations:** 1 Research Institute of Child Development and Education, University of Amsterdam, Post Box 15776, 1001 NG Amsterdam, The Netherlands; 2 Clinical Child and Family Studies, Utrecht University, Post Box 80140, 3508 TC Utrecht, The Netherlands

**Keywords:** Home visitation program, Child maltreatment prevention, Increased effect, Parental sense of competence, Perceived stress, Parental anger, PTSD symptoms

## Abstract

**Purpose:**

Home visitation program effects are generally small, which may be caused by flexible intervention content leading to inconsistent outcomes. In this study we therefore examined whether the effectiveness of a Dutch home visitation program (i.e., Supportive Parenting) can be improved by adding structured intervention components targeting key risk factors for child maltreatment: parental sense of competence, perceived stress, parental anger, and PTSD symptoms.

**Method:**

Participants were randomly assigned to an experimental group (*n* = 74) that received four additional intervention components in two home visits, or a control group (*n* = 60) that received regular Supportive Parenting. Outcomes were assessed before (T1) and after (T2) the first, and before (T3) and after (T4) the second home visit. Effects were examined using ANCOVA for primary outcomes: parental sense of competence, perceived stress, parental anger, and PTSD symptoms, and secondary outcomes: risk of child maltreatment, parental warmth, and negative parenting. Moderation effects were examined for T1 scores, child temperament and life events.

**Results:**

Mothers who received the intervention components showed less stress compared to the control group at T3 and T4. There were no differences between groups on other outcomes and no moderation effects, although parental sense of competence reduced and anger increased within the experimental group specifically.

**Conclusion:**

The structured components may enhance the effectiveness of Supportive Parenting to reduce parenting stress. Future research into how other outcomes can be improved is needed.

**Supplementary Information:**

The online version contains supplementary material available at 10.1007/s10896-023-00509-7.

Given the prevalence and severe negative consequences of child maltreatment (Moody et al., 2018), it is essential that established prevention programs invest in continuously improving their effectiveness. On average, child maltreatment prevention programs have limited effects (Van der Put et al., 2018), which leaves room for improvement. The present study therefore tests whether a widely disseminated home visitation program to prevent child maltreatment in the Netherlands can be improved by adding structured intervention components targeting four risk factors for child maltreatment: parental sense of competence, perceived stress, parental anger, and PTSD symptoms.

Home visitation programs can prevent child maltreatment, although effects are generally small (Casillas et al., 2016). One feature often valued and hypothesized to contribute to home visitation effects is flexibility in intervention contents (Azzi-Lessing, 2011). This means that professionals have the freedom to decide which techniques and timing they will use to support the family, ensuring a personalized intervention program for every family. This flexibility, however, comes at the cost of ensuring that all families receive evidence-based techniques known to contribute to intervention effects (see e.g., Chaffin & Friedrich, 2004).

In the present study we tested whether “combining the best of both worlds” – adding specific structured intervention techniques to a flexible home visitation program in the Netherlands – increases program effects. Supportive Parenting (SP; in Dutch “Stevig Ouderschap”, see Bouwmeester-Landweer, 2006) is part of care as usual in 53% of municipalities in the Netherlands. Families living in these regions receive a survey prenatally or right after childbirth to screen for risk factors of child maltreatment using a screening instrument (e.g., history of child maltreatment, or substance abuse; Van der Put et al., 2017). Families scoring above the cut-off are offered the SP program consisting of home visits by a qualified youth health care nurse roughly every 3 months in the first year and every 6 months in the second year until the child is up to 2 years old. Although the program includes several fixed topics (e.g., parents’ own developmental history, parenting perception and expectations, and social and professional support), the program contents are generally flexible and various intervention techniques may be used.

The SP program has shown small to large positive effects on improving parental expectations, improving child development, increasing successful referral to psychological care, and increasing early detection of child maltreatment in an early randomized controlled trial (Bouwmeester-Landweer, 2006). Notably, this study found 22% of the control group versus 8% of the experimental group at risk for child maltreatment after the intervention, and overall improvement in six to nine outcome measures for 10% of the control group and 18% in the experimental group. However, a more recent study found no differences on parenting skills, social support, the parents’ self-sufficiency and resilience, and the child’s psychosocial health and behavior (Van Grieken et al., 2019), but this lack of effects may be due to several limitations in the study design, such as a non-random control group. While the intervention targets both mothers and fathers, mothers are mostly present during SP home visits (Bouwmeester-Landweer, 2006), and we therefore focus specifically on mothers in this study.

We test the added effects of four intervention components targeting four key risk factors for child maltreatment: lack of parental sense of competence, perceived stress, parental anger, and Post Traumatic Stress Syndrome (PTSD) symptoms. First, a lack of parental sense of competence may lead a mother to give up sooner on difficult parenting tasks, due to a lack of belief that her parenting behavior will be effective (Jones & Prinz, 2005), sooner resorting to inadequate parenting behavior such as harsh and inconsistent parenting practices (Sanders & Woolley, 2005). Parental sense of competence can be improved using social learning theory principles such as social comparison and positive feedback, in ways that spill-over to more positive parenting practices (Mouton & Roskam, 2015) and may therefore decrease the risk for child maltreatment. Indeed, child maltreatment programs that include components targeting parental self-efficacy have shown to be more effective in reducing the risk of child maltreatment than programs without such a component (Van der Put et al., 2018).

Second, perceived stress increases the risk of child maltreatment (Rodriguez & Richardson, 2007) through increased risk for harsh parenting (Beckerman et al., 2018). Persistent high levels of stress and difficulties to adequately cope with this stress may compromise parental resiliency which may lead to inadequate parenting practices such as spanking (Martorell & Bugental, 2006). Therefore, increasing stress coping skills to reduce perceived stress may increase parental resiliency to adopt adequate parent practices in difficult parenting situations. Furthermore, programs including intervention components that target personal skills such as stress management skills have been the most effective among child maltreatment prevention programs (Van der Put et al., 2018).

Third, maltreating parents typically find it more difficult to manage feelings of anger than non-maltreating parents (Lavi et al., 2019), and abuse is sometimes a consequence of a lack ability to cope with anger in non-violent ways (Rodriguez, 2010). One source of anger can be dysfunctional attributions about the child’s responsibility for his or her behavior (e.g., that children cry to nag their parents; Pidgeon & Sanders, 2009). Therefore, targeting negative attributions may help parents reduce feelings of anger and increase appropriate coping skills, thereby reducing the risk of child maltreatment. This approach has already proven effective in a parent group training setting (Sanders et al., 2004), and may generalize to home visiting programs as well.

Fourth, PTSD symptoms may lead to deficits in maternal emotion regulation (Kumar et al., 2020), which makes it difficult to parent assertively and adequately, and may lead to a less positive and affectionate mother-child relationship (Van Ee et al., 2016), and harsher parenting (Ammerman et al., 2012), increasing the risk for child maltreatment (Banyard et al., 2003). Consequently, adequate recognition and referral to treatment of PTSD symptoms may enhance the effects of programs to prevent child maltreatment (Ammerman et al., 2012).

In this study, using a randomized controlled trial, we examined whether mothers who received four manualized intervention components in addition to standard home visiting show increased parental sense of competence, and decreased perceived stress, parental anger, and PTSD symptoms, compared to a control group of mothers who received standard home visiting only. We expected these components to reduce the risk of child maltreatment and improve parenting practices. Because the added intervention components may not be effective for all mothers in the same way, we examined several possible moderator effects. First, mothers that have better scores on the targeted risk factors at pretest, may already harbor more skills to be able to engage in the program content (Wagner et al., 2003), thereby increasing the potential positive intervention effects. On the other hand, mothers experiencing more difficulties at pretest may have more room for improvement (Muzik et al., 2015). We therefore tested whether baseline scores on the four targeted risk factors moderate the effects of the intervention components. Second, and similarly, mothers of children with more difficult temperament may have more difficulty applying the intervention techniques in practice, because their child’s temperament may make parenting more challenging (Hipke et al., 2002), or may benefit more because they are at the most need of additional intervention techniques (Anzman-Frasca et al., 2014). Finally, mothers living in more turbulent times due to many important life events (e.g., moving, losing a loved one) may be too preoccupied with processing those events to properly engage in the intervention components (Klebanov et al., 2001) and benefit less, or benefit more if the intervention components aid in adequately processing these life events (Kersten-Alvarez et al., 2010). In sum, we explored baseline risk factor scores, child’s temperament, and the number of recent life events as possible moderators of the effects of our added intervention components, without a priori hypotheses about the direction of such effects.

## Methods

### Trial Design

Data for this trial, using equal randomisation based on a computerized random number generator, were collected between March 2019 and August 2021 in the Netherlands. The experimental group received SP + additional intervention components; the control group received regular SP.

Mothers completed four online questionnaires around two consecutive SP home visits A and B: two weeks before home visit A (T1), two weeks after home visit A (T2), two weeks before home visit B (T3), and two weeks after home visit B (T4). Nurses filled in a checklist about the contents of the home visit right after each home visit (Checklist A and B). Nurses and mothers were aware of allocation to experimental versus control condition, but mothers were unaware of the study’s hypotheses. The study protocol for this trial was published open access (De Wit et al., 2020).

### Participants

Nationwide, 11 organisations participated, and 93 qualified SP youth health care nurses were trained to deliver the additional intervention components. For nurses trained in the first study year, an online booster training was provided in the second year (*n* = 30). Nurses asked eligible mothers in their caseload to participate, and then the mother was contacted by one of the researchers and asked for informed consent. Mothers were eligible for participation if they had at least two postnatal SP home visits left and had sufficient understanding of the Dutch or English language. Mothers received a gift voucher of €5 per questionnaire, with an additional €5 if they completed all questionnaires (total € 25). Nurses received a gift voucher of €5 if mother completed at least T1 and T4. Originally, the study aimed to include 398 participants, but the COVID-19 pandemic made recruitment challenging for nurses. Participant inclusion was ended at *n* = 134 in January 2021.

After inclusion ended, we examined whether there was any selection bias, and the outcomes are reported in Appendix A. This Appendix shows that we were able to reach over half of the originally trained nurses (*n* = 53, 57%). Although these non-participant data were therefore not complete, they show that the reason for not being invited by the nurse to participate lies in complex, multi-problem cases in which the nurse was already struggling to apply SP alone. In addition, the majority of the mothers declined because they stated that they had too much going on in their life. Both could indicate that our study may have not reached the more multi-problem or complex SP participants, making our outcomes less representative of those participants. Furthermore, it is unclear how the COVID-19 pandemic played a role in the reasons for nurses not to ask for participation, or for mothers to decline participation.

### Interventions

#### Supportive Parenting

SP is a nurse home visiting intervention in the Netherlands (see Bouwmeester-Landweer, 2006). The intervention is intended for parents with an increased risk for parenting problems and child maltreatment, based on a postnatal risk screening instrument (i.e., Instrument for early Identification for Parents At Risk for child Abuse and Neglect, IPARAN; Horrevorts et al., 2017; Van der Put et al., 2017), led by the professional assessment of a youth health care nurse. The intervention consists of six to ten postnatal 90-minute home visits by a qualified youth health care nurse and aims to prevent severe parenting problems, including child maltreatment, for children up to 2 years old. The home visits cover the following topics: parenting experiences, managing the parents’ own developmental history, expectations about the child’s development and the family’s social (and professional) support system. Around these subjects, the contents of the visits are flexibly adapted to the parents’ preferences and needs using a combination of intervention techniques, such as: motivational interviewing, health education, parenting advice, positive reinforcement, observational techniques, modelling, and registration techniques and/or homework assignments (Bouwmeester-Landweer, 2017).

Adherence to the intervention protocol is ensured through a four-day initial training for nurses, followed by mandatory supervision of at least six meetings a year in the first two years of practicing the SP intervention. In addition, nurses are expected to guide a minimum number of families per year, and regularly attend supervision and relevant training courses to maintain their SP license. Finally, the providing organisations are audited about program adherence every five years. However, intervention adherence for the actual participants in the present study was not measured.

#### Intervention Components

We designed four intervention components to reduce key risk factors for child maltreatment: increasing parental sense of competence, reducing perceived stress, reducing parental anger, and identifying PTSD symptoms. The theoretical underpinnings have been described by De Wit et al. (2020).

First, to increase parental sense of competence, nurses gave structured positive feedback about parenting practices, including four components: a description of the observed behavior, a comparison with other mothers, an explanation of the positive effect of the behavior, and a value label. This study required positive feedback in at least two occasions per home visit (i.e., A and B). Second, to reduce perceived stress, the nurse introduced mothers to a 10-minute guided imagery audio relaxation exercise. After listening to the exercise together in the first home visit (A) and discussing daily use of the exercise, the use of the exercise was evaluated and once more encouraged in the second home visit (B). Third, to reduce parental anger, the nurse normalized angry responses to difficult child behavior, how they can be recognized, and introduced techniques to help parents calm down when they feel angry. Next, the nurse discussed flash cards on which common anger-evoking situations and possible dysfunctional attributions as well as adequate attributions and behaviors were described. These subjects were all discussed in home visit A. In home visit B, the contents of visit A were evaluated, and the nurse and mother discussed 1–2 empty flash cards with an individualized anger-evoking situation. Finally, to identify symptoms of PTSD, the nurse used the two-item version of the abbreviated PTSD Checklist – Civilian (PCL-C; Lang & Stein, 2005) to assess the level of mothers’ PTSD symptoms and – if applicable – encourage mother to seek professional treatment for these symptoms in home visit A, and evaluated this in home visit B.

Intervention fidelity was tracked by examining nurses’ checklists, and these outcomes are shown in Appendix B. First, there were no significant differences between groups in percentage of home visits delivered in person versus phone/video call, although some home visits did not take place at the parents’ home, mainly due to COVID-19 restrictions. Next, we examined how often the subjects of the additional intervention components were generally covered. We found that positive feedback, stress, and anger were just as often subject of discussion for the control group as for the experimental group, indicating that the intervention components did not introduce any new interventions subjects, but only new techniques. However, trauma was significantly less often a subject of discussion in the control group, compared to the experimental group. The added intervention techniques may have provided a concrete reason for starting a hard to discuss topic.

Execution of the added intervention components was moderately successful in terms of intervention fidelity. The components targeting sense of competence, stress and PTSD symptoms were implemented sufficiently based on the criterion by Durlak and Dupre (65–70% in at least one home visit; i.e., > 60%, Durlak & DuPre, 2008), although we originally targeted implementation in both home visits (implemented 22–27%). Unfortunately, implementation of the anger component did not meet this criterion (i.e., implemented for 12% in both, and 39% in at least one home visit). However, about halfway during the study (i.e., August 2020) we found a technical fault in a part of the survey concerning implementation of the anger component. This was corrected once we found out but may have been the cause for the reported substantial difference between implementation of this and other components.

Appendix B also shows there was some contamination of intervention techniques into the control group. A proportion of control group participants received the additional intervention techniques in both (i.e., 2–10%) or in at least one home visit (i.e., 20–33%). However, there still was a significant difference between application of the techniques between the experimental and control group for the sense of competence, stress, and PTSD components. Interestingly, mothers would use the offered relaxation exercise significantly less often in the control group compared to the experimental group, which may indicate that the exercise was initiated less extensively in the control group. In addition, when the PTSD checklist was used, similar proportions of mothers scored above the threshold and of them, similar proportions were prepared to seek help, regardless of experimental group. Only for the anger component the proportion participants that received the intervention techniques in at least one home visit were similar, and it is unclear how the abovementioned technical fault has played a role in this similarity. Differences in outcome based on potential effects of the anger component may therefore have been more difficult to detect.

### Measures

Primary outcomes were the risk factors targeted by the four intervention components: parental sense of competence, perceived stress, parental anger, and PTSD at T2, T3, and T4. Secondary outcomes were more distant outcomes at T4: the risk of child maltreatment and parenting behavior. Finally, we measured putative moderators at T1: child temperament and life events in addition to demographics, such as age and ethnicity.

#### Parental Sense of Competence

We used the shortened Sense of Competence subscale of the Parenting Stress Index (PSI; Abidin, 1983; De Brock et al., 1992). Nine items, such as “I feel that I am not very good at being a parent”, were answered on a six-point Likert scale ranging from 1 (*I totally disagree*) to 6 (*I totally agree*) and were reverse coded so that higher scores reflect a higher level of parental competence. This measure showed excellent reliability with Cronbach’s alphas ranged *α* = 0.91-0.93 between time points.

#### Perceived Stress

We used the Perceived Stress Scale (PSS-10; Cohen et al., 1983; Cohen, 1988). The items such as “In the last month, how often have you found that you could not cope with all the things you had to do?” were rated on a five-point scale ranging from 1 (*never*) to 5 (*very often*) with higher scores reflecting higher levels of perceived stress. Reliability of this measure was acceptable across all assessment points: *α* = 0.76-0.80.

#### Parental Anger

We used the ‘expression’-subscale of the Parental Anger Scale (PAS; Gavita et al., 2011). Using items such as “I get so angry with my child, that I scream or yell at my child”, anger was scored on a seven-point scale ranging from 0 (*never*) to 6 (*several times a day*) with higher scores reflecting more anger. Parental anger showed acceptable to good reliability *α* = 0.75-0.83.

#### PTSD Symptoms

We used the abbreviated PTSD checklist civilian (PCL-C; Lang & Stein, 2005), which consist of six items that ask about the occurrence of PTSD-symptoms such as “repeated, disturbing memories, thoughts, or images of the stressful experience”. The instrument measures on a five-point scale ranging from 0 (*not at all*) to 4 (*extremely*), and scores of 3 or higher are considered symptomatic. This measure showed acceptable reliability, range *α* = 0.70-0.77 at all assessment points.

#### Risk of Child Maltreatment

We used the Instrument for early Identification for Parents At Risk for child Abuse and Neglect (IPARAN; Horrevorts et al., 2017; Van der Put et al., 2017). This instrument consists of 16 questions on the presence of the most important risk factors for child abuse, such as “I can get so angry that I lose control”. Nine items are answered on a four-point scale ranging from 1 (*always*) to 4 (*never*), and seven items are yes/no items. The answer options have been scored between 0 and 2 in line with their weight in predicting the risk of child maltreatment, with higher scores representing a higher risk of child maltreatment. Reliability for this measure could be considered sufficient at T1 (i.e., *α* = 0.57) and T4 (i.e., *α* = 0.68), given the homogeneity of the construct under investigation (see Streiner, 2003).

#### Parenting Behavior

We used the rejection, hostility, attention, and affection subscales of the Comprehensive Parenting Behavior Questionnaire (CPBQ; Majdandžić et al., 2008; Majdandžić et al., 2016). We aggregated this measure into two dimensions of parenting: Negative parenting (i.e., rejection and hostility) and Warmth (i.e., attention and affection). The 14 items such as “Sometimes I am really fed up with my child, and this clearly shows” (Rejection subscale), or “I regularly play or talk with my child for at least 5 min, with our attention focused on each other, just for fun” (Attention subscale) are rated on a five-point scale ranging from 1 (*totally not applicable*) to 5 (*completely applicable*). Higher scores represent more positive or more negative parenting. Cronbach’s alphas were acceptable for Negative parenting at T1 and T4 and Warmth at T1 (i.e., *α* = 0.74-0.77) and good for Warmth at T4 (i.e., *α* = 0.84).

#### Moderators

We measured two dimensions of child temperament: soothability and negative emotionality of the Revised Infant Behavior Questionnaire (IBQ-R; Gartstein & Rothbart, 2003). The soothability subscale consisted of 18 items such as “when singing or talking to your baby, how often did s/he soothe immediately”. For negative emotionality, 12 items such as “at the end of an exciting day, how often did your baby become tearful” from the Very Short Form of the IBQ-R (IBQ- R VSF; Putnam et al., 2014) were used. The original items use a seven-point scale ranging from 1 (*never*) to 7 (*always*), but we used four of the original answer categories for both scales (*almost never, less than half of the time, more than half of the time, almost always*) to accommodate for mothers with lower educational levels. Higher scores represented easier temperament for soothability, and more difficult temperament for negative emotionality, and reliability could be considered questionable for soothability (i.e., *α* = 0.69), and good for negative emotionality (i.e., *α* = 0.85).

The occurrence of life events in the past 12 months was measured using the “life events”-subscale of the PSI (Abidin, 1983; De Brock et al., 1992). This subscale consists of thirty yes/no-items such as “I got fired/I quit my job” or “I had a miscarriage”. We used the total number of (different) life events as a moderating variable in our analyses.

### Analytical Methods

First, we have conducted multiple imputation procedures to be able to include data for all participants in all analyses using chained equations (i.e., MICE in R; Van Buuren & Groothuis-Oudshoorn, 2011; Van Buuren, 2018). We tested for missing data patterns using Little’s missing completely at random test, which indicated that data was missing completely at random (χ^2^ = 445, *p* = .465).

Predictors for imputation were experimental group, participant demographics (i.e., maternal age, education, other children, care situation, financial situation, ethnic background, baby age, other support, living situation), moderators (i.e., life events, negative emotionality and soothability), and all outcome measures at T1, T2, T3, and T4. As advised by Van Buuren (2018), we conservatively set the number of imputations and iterations to the maximum proportion of missing data in the study, which was 40% at T3. This relatively large proportion of missing data was often because there was not enough time between T2 and home visit B to conduct T3. Substantially fewer missing values were observed at other time points (i.e., 16% for maternal age, 1% for all other demographics and for moderators at T1; outcomes: T1 = 5%, T2 = 8%, T4 = 4%).

Intervention effects on our primary and secondary outcomes have been examined using pooled analysis of covariance (ANCOVA) with baseline scores (T1) as the covariates, with data of T2, T3, and T4 as dependent variables. To test who benefits most from the manualized components we have tested whether condition × pretest score, children’s temperament (i.e., negative emotionality and soothability) and the number of life events in the year before T1 moderated the effects on the primary and secondary outcomes at T4. We conducted sensitivity analyses to test whether there were any differences in outcomes between the original and imputed data but found no differences and therefore only reported outcomes based on the imputed dataset. Finally, we conducted post-hoc paired t-tests to examine within-group differences between T1 and T4 for all primary and secondary outcomes for the experimental and control group separately.

### Deviations from the Study Protocol

In the present manuscript, we deviated from the study procedures we described in our study protocol (i.e., De Wit et al., 2020) in three ways. First, we did not include a second control group consisting of *n* = 25 mothers with nurses from a SP organisation who did not receive training in the additional intervention components. Due to disappointing participant numbers, in particular during the COVID-19 pandemic, the additional group was omitted from the study. Second, due to the applied multiple imputation method for missing values (i.e., multiple imputed datasets), we were not able to use MANCOVA analyses, because the results of those analyses cannot be pooled. Instead, we used ANCOVA for all outcome effect analyses. Also, the within-group analyses were determined post-hoc. Finally, we did not conduct any mediation analyses examining (the changes in) the main intervention outcomes, because our analyses showed no significant effect for intervention group on the risk of child maltreatment, and this main effect is conditional for mediation analyses (Baron & Kenny, 1986).

## Results

### Participant Flow

**Fig. 1:**
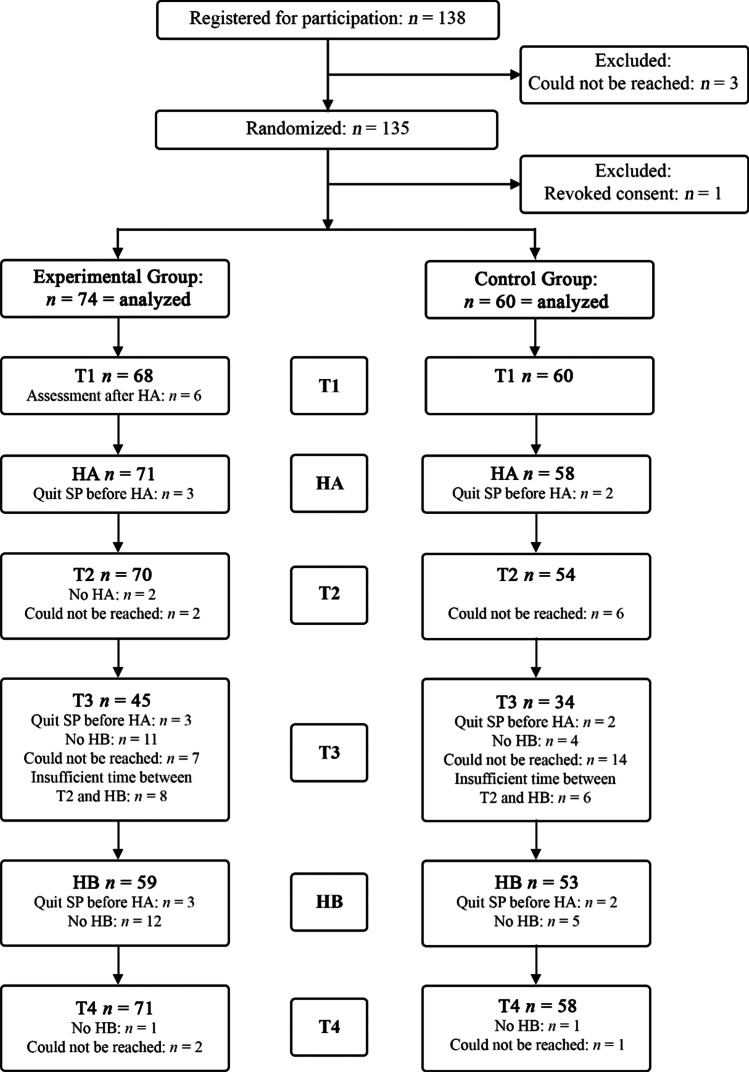
Participant flow. *Note.* HA = Home visit (A) HB = Home visit (B) SP = Supportive Parenting

Participants were included for T1 from March 2019 until December 2020; T4 assessments took place until August 2021. Figure 1 depicts the participant flow throughout the study. The Figure shows that *n* = 134 mothers; *n* = 74 in the experimental group (EXP), and *n* = 61 in the control group (CTRL), completed at least one assessment point. For six mothers in the experimental group the T1 assessment took place after home visit A, therefore only their demographic and moderator scores were retained and their T1 outcome scores were handled as missing. Furthermore, *n* = 5 mothers (EXP *n* = 3, CTRL *n* = 2) did not get a home visit A, and *n* = 22 did not get a home visit B (EXP *n* = 15, CTRL *n* = 7), but their assessments were – where feasible – treated as if they did get a home visit, in line with an intent-to-treat approach. All mothers were contacted for the T4 assessment, either after home visit B, or when there would not be another home visit before the end of the research period. Due to the use of multiple imputation, data from all mothers could be used for analyses.

The number of days between assessment points and home visits is shown in Appendix C. While we aimed for all assessments to be 2 weeks before or after the home visits, and about 3 months between home visits, in practice, there were on average between 15 and 19 days between the home visits and the assessments points, and 73 days between the home visits. However, there was only a significant difference in days between the experimental and control group between the T3 and T2 assessments: in the control group, T3 was sooner after T2 than in the experimental group. In addition, compared to T1, only home visit B and T4 were sooner after T1 in the control group than in the experimental group. This could indicate that experimental group mothers were able to manage alone for a longer period than control group mothers due to larger positive effects of the first home visit. However, – with no clear outcome pattern to confirm this hypothesis – it could also indicate that there were differences between groups other than just the additional intervention components.

### Participant Characteristics

Table 1 shows the baseline characteristics of the participants per group. Participating mothers were on average 29 years old and their babies on average 6 months old. Most mothers finished at least higher general secondary education. For most of the mothers, the child they received SP for was their first child, and most did not receive any other help or counselling, were born in the Netherlands just as their parents, and were living with and sharing care for the baby with the baby’s father. Furthermore, mothers had experienced 1.5 life events in the past year. There was a significant difference between groups in the financial circumstances: only in the experimental group some mothers reported that they hardly had enough money to make ends meet, while all other participants reported that they mostly, or always had enough money to make ends meet. Notably, a larger proportion of participants in the experimental group also reported that they always had enough money to make ends meet, compared to the control group. There were no significant differences between the groups on any of the other baseline characteristics.

**Table 1:** Baseline characteristics for the experimental and control group

	EXP		CTRL			
	*M*/*n*	*SD*/*%*	*M*/*n*	*SD*/*%*	t/*χ*^*2*^	*p*
Age	29.77	4.83	29.66	5.00	− 0.13	0.896
Age baby (months)	6.51	3.69	6.05	4.68	− 0.64	0.523
Education level					1.23	0.296
Elementary school	4	5	0	0		
Lower general secondary education	8	11	6	10		
Higher general secondary education	31	42	33	56		
University (of applied science) bachelor or master	30	41	20	34		
Other	1	1	0	0		
Other children					0.92	0.399
No	50	68	35	58		
1 other child	18	24	21	35		
> 1 other children	6	8	4	7		
Receiving other help/counselling					0.34	0.559
No	51	69	45	75		
Yes	23	31	15	25		
Mother and both parents born in the Netherlands					0.11	0.743
No	56	76	43	72		
Yes	18	24	17	28		
Shared Care with others					0.86	0.464
Yes, with the child’s father	66	89	51	85		
Yes, with another partner	0	0	3			
Yes, with someone else	1	1	1	2		
No	7	9	6	10		
Having enough money to make ends meet					5.16	0.006**
Hardly	5	7	0	0		
Mostly	13	18	23	38		
Always	56	76	37	62		
Living situation					0.15	0.981
Alone	11	15	8	13		
With the child’s father (and other children/parents/in-laws)	60	80	48	82		
With another partner	2	3	2	3		
With parents/in-laws (and siblings)	1	1	1	2		
Life Events	1.55	1.26	1.50	1.55	− 0.22	0.824
*Temperament*						
Negative Emotionality	3.08	0.84	3.29	0.95	1.34	0.182
Soothability	5.10	0.77	5.02	0.61	− 0.68	0.495

*Note.* EXP = Experimental group. CTRL = Control group

* *p* < .05. ** *p* < .01. *** *p* < .00

Baseline (T1) scores on the primary and secondary outcomes are reported in Table 2. There were no significant differences at T1 between the experimental and control group in parental sense of competence, perceived stress, parental anger, PTSD symptoms, risk of child maltreatment and parental warmth. However, at T1, the mothers in the control group did show more negative parenting than mothers in the experimental group.

**Table 2:** Differences between groups on T1, T2, T3, T4 on primary and secondary outcomes

	EXP		CTRL				
	*M*	*SD*	*M*	*SD*	*t/F*	*p*	*d*^*+*^
	T1						
Primary outcomes							
Parental Sense of Competence	17.91 (8.98)		19.12 (8.33)		0.80	0.426	− 0.139
Perceived Stress	17.49 (5.59)		18.56 (6.16)		1.06	0.293	0.183
Parental Anger	0.20 (0.28)		0.32 (0.42)		1.88	0.062	0.343
PTSD Symptoms	12.89 (4.00)		12.76 (4.67)		− 0.18	0.857	− 0.030
Secondary outcomes							
Risk of Child Maltreatment	3.20 (2.62)		3.55 (2.74)		0.76	0.447	0.131
Parental Warmth	4.62 (0.37)		4.51 (0.45)		-1.56	0.122	0.270
Negative Parenting	1.46 (0.46)		1.68 (0.60)		2.45	0.015*	0.417
	T2						
Primary outcomes							
Parental Sense of Competence	17.75 (9.78)		18.98 (8.90)		− 0.19	0.851	0.008
Perceived Stress	16.44 (6.43)		18.58 (6.22)		-1.53	0.130	0.155
Parental Anger	0.25 (0.34)		0.35 (0.41)		− 0.28	0.781	− 0.075
PTSD Symptoms	11.62 (4.14)		12.32 (4.93)		-1.16	0.248	0.185
	T3						
Primary outcomes							
Parental Sense of Competence	16.28 (8.35)		18.38 (8.90)		-1.00	0.319	− 0.105
Perceived Stress	15.95 (5.99)		19.07 (5.74)		-2.43	0.018*	0.348*
Parental Anger	0.27 (0.47)		0.35 (0.44)		0.16	0.871	− 0.168
PTSD Symptoms	11.66 (3.58)		12.02 (4.31)		− 0.70	0.484	0.122
	T4						
Primary outcomes							
Parental Sense of Competence	16.42 (8.27)		18.97 (9.28)		-1.64	0.104	− 0.153
Perceived Stress	15.79 (5.35)		18.22 (5.87)		-2.37	0.019*	0.252*
Parental Anger	0.28 (0.39)		0.34 (0.47)		1.02	0.311	− 0.140
PTSD Symptoms	12.13 (3.93)		12.31 (4.25)		− 0.46	0.643	0.074
Secondary outcomes							
Risk of Child Maltreatment	3.23 (2.99)		3.65 (3.06)		0.31	0.758	0.008
Parental Warmth	4.61 (0.43)		4.50 (0.53)		0.25	0.802	− 0.039
Negative Parenting	1.53 (0.52)		1.70 (0.57)		− 0.06	0.951	− 0.104

*Note.* EXP = Experimental group. CTRL = Control group

^+^ = Corrected for pre-test at T2, T3, and T4

* *p* < .05. ** *p* < .01. *** *p* < .001

### Primary Outcomes

The primary outcomes were measured at T2, T3, and T4. Differences between groups were compared at each assessment points using ANCOVA, with the pretest score of that outcome as a covariate. The results for these analyses can be found in Table 2, and outcome graphs are included in Appendix D.

There were only significant differences between the experimental group and control group for perceived stress at T3 and T4. After one and two home visits respectively, mothers in the experimental group showed less stress than mothers in the control group. This difference was not (yet) significant at T2. For the other outcomes: parental sense of competence, parental anger, and PTSD symptoms, no significant differences between groups were found at T2, T3 and T4.

### Secondary Outcomes

Secondary outcomes were assessed at T1 and T4 and included the risk of child maltreatment, parental warmth and negative parenting as shown in Table 2, and outcome graphs are included in Appendix D. There were no significant differences between groups at T4 on any of the secondary outcomes.

### Moderators

Moderating effects were examined for all outcomes (both primary and secondary) at T4, using moderators measured at baseline. Included as moderators were T1 scores, child temperament (i.e., negative emotionality and soothability) and life events. No significant moderating effects were found for any of the moderators on any of the primary of secondary outcomes.

### Within-Group Changes from T1 to T4

To aide in the interpretation of these outcomes and graphs, we conducted post-hoc analyses to examine the differences between pre- and posttest, i.e., T1 and T4 within each group. These analyses showed there were significant differences between pre- and posttest for the experimental group on parental sense of competence, perceived stress, and parental anger. That is, mothers showed less parental sense of competence (*t* = 2.07, *p* = .042), less perceived stress (*t* = 3.16, *p* = .002), and more parental anger (*t* = -2.42, *p* = .018) after the two experimental home visits. Control group mothers experienced no significant changes on these outcomes during the study. No significant differences between T1 and T4 were found for the experimental or control group for PTSD symptoms, the risk of child maltreatment, parental warmth, and negative parenting.

## Discussion

The present study tested whether a home visitation program to prevent child maltreatment in the Netherlands improved by adding structured intervention components targeting four risk factors for child maltreatment: parental sense of competence, perceived stress, parental anger, and PTSD symptoms. Using a randomized controlled trial, we compared 74 mothers who had received the home visiting program SP + additional intervention components to 60 mothers who had received standard SP on four primary outcomes: parental sense of competence, perceived stress, parental anger, and PTSD symptoms, and three secondary outcomes: risk of child maltreatment, parental warmth, and negative parenting. Mothers who received the additional intervention components showed significantly less stress than mothers in the control group at T3 and T4. There were no significant differences between groups on the other primary or secondary outcomes and no moderation by child temperament or life events.

These findings suggest that the stress-component can enhance the effects of SP. The hypothesized key element of the stress-component is a relaxation exercise that mothers are asked to use frequently. However, less than a third of mothers reported using the exercise (i.e., 31%). It might be that some of the other elements of the stress-component (e.g., normalizing stress) as well as elements of other components (e.g., discussing ways to calm down in the anger component) may have contributed to stress reduction. This is in line with a previous study that found similar stress reduction for an experimental group of mothers that received a relaxation-training program when compared to a control group that received a relaxation education program (i.e., using an exercise versus psychoeducation; Chuang et al., 2012; Fotiou et al., 2016). In addition, studies that did find effects of relaxation exercises on parental stress most often studied supervised relaxation exercises, leaving the influence of self-initiated daily exercising unclear (see e.g., Dabas et al., 2019; Tsitsi et al., 2017).

The lack of intervention effect on the other outcomes can be explained in several ways. First, although intervention fidelity was sufficient for the use of the intervention components in at least one of the home visits (65–70%; i.e., > 60%, Durlak & DuPre, 2008), it was low for the originally anticipated use in both the home visits (i.e., 12–32%). Thus, mothers may have not received a sufficient dose of the components to benefit. In addition, there was some contamination between conditions such that some mothers in the control group unintendedly received the additional intervention components (20–33% in at least one, and 2–10% in both home visits). This might further mask possible effects of the additional components, although consequences may have been limited, because both intervention groups hardly showed any changes. Second, we were able to include fewer participants than originally planned (i.e., N = 398, see De Wit et al., 2020), which has reduced statistical power to find the relatively small effects we anticipated. Third, for this study the additional intervention components consisted of fixed intervention techniques in two fixed home visits. This may have undermined the hypothesized effects of flexible application of intervention techniques within the normal course of SP, and thereby have diminished the potential effects of additional intervention components. Fourth, several characteristics of our outcome measures may have hindered detecting intervention effects. Our measure for parental anger showed little variability around a low mean, which may indicate that either anger was generally low in this sample, or that mothers underreported their parental anger. However, in spite of the limited variation, only the experimental group experienced a significant increase in anger between T1 and T4, which is hard to explain due to uncertainties about the actual application of the anger component. Both make it difficult to identify benefits or disadvantages of the components for mothers who experience parental anger. Furthermore, our measure for PTSD symptoms measured these symptoms only, while the intervention component targeting maternal trauma mainly focuses on increased referral to treatment. Although the nurses indicated that the majority of referred mothers would be motivated for treatment, the effects of this treatment would not be expected to show within the duration of the study. Therefore, it may not have been realistic to use the outcome of PTSD symptoms to measure effects of this intervention component. A different outcome, measuring the number mothers that started treatment because of the intervention component, or measuring symptoms at a considerably longer follow-up, would be more appropriate to measure the effects of this intervention component.

Importantly, levels of parental sense of competence developed opposite to what we expected. That is, the experimental group showed a reduction, rather than an increase, in parental sense of competence; parents felt less competent at each assessment point compared to the previous, even though this did not lead to a significant difference between the experimental and control group at any time point. This pattern, however, may indicate a negative, detrimental effect of the added intervention components. This would not be in line with the elaborate research base documenting the positive effects of positive feedback (e.g., Mouton & Roskam, 2015). The present outcomes may indicate the so-called Dunning-Kruger effect, which states that competence correlates with the ability to self-assess (Kruger & Dunning, 1999). In this case: parents who know less about parenting skills will rate their competence higher (because they do not know what they do not know) than parents that have more knowledge about parenting skills (because they are aware that their parenting is not fully in line with some of the recommendations). If this were true, then the added intervention components may have increased parenting knowledge, facilitating parents to be more critical about their parental competence. Future research is needed to test whether this is indeed the case, or whether some characteristics of the intervention worked to undermine mothers’ feelings of parental competence.

Arguably, if the outcomes on parental sense of competence, parental anger and PTSD symptoms are less reliable in the outcome effects we aimed to measure, because they show unexpected and undesirable patterns, then why would the outcome of parental stress be any more valid? First, this outcome consisted of the most items to measure – arguably – the most unidimensional construct of all outcomes, which increases confidence in this outcome measure. Second, we believe that perceived stress as a construct is more variable over time, and outcome scores will therefore be a reflection of a temporary mood, rather than more stable internalized thoughts and feelings with constructs such as sense of competence, parental anger, and PTSD symptoms. This is reflected in the perceived stress outcome graph shown in Appendix D, that shows more variability over time than the other outcomes. Consequently, effects of intervention components to improve this outcome, would also reflect in outcome measure the quickest, while other outcomes may have taken longer to reflect the changes that we aimed for in the current study.

Generally, this study shows that there is still much room for improvement in the feasibility of both the intervention and the additional components. That is, some families were not included in this study because the nurses were already struggling to apply regular SP, while several nurses in the experimental struggled to implement (parts of) the additional components. Although it would not be unreasonable to expect that SP – as a secondary preventive intervention – functions as a gateway to reveal more serious problems for some cases, and a steppingstone for referral to a broader system of care for other cases, a high number of those cases may warrant improvement in the indication process for this intervention. Furthermore, if we want to improve this intervention in daily practice, then additional research is needed to determine the conditions under which this intervention and the additional components are the most feasible and effective.

Although the present study provides valuable new insights into the merit of adding manualized intervention components to home visiting interventions in the prevention of child maltreatment, some limitations need to be mentioned. First, we conducted this study partly during the COVID-19 pandemic and SP was not always conducted as prescribed during this time. For instance, the number of home visits by primary health care nurses was limited during lockdowns (i.e., the Netherlands had lockdowns in the spring of 2020 and the winter of 2020/2021) and this may have influenced both the dosage and quality of the intervention. Moreover, adherence to the intervention was not measured, while fidelity to the additional components only consisted of limited information derived from one source (i.e., nurses). Therefore, it cannot be ruled out that the effectiveness of both the existing intervention as well as the additional components understates the actual effects due to a lack of intervention adherence. Second, there may have been a selection effect in that mothers who received SP but who did not want to participate in this study may have shown more severe problems than mothers that did participate. This may have limited our ability to detect intervention effects of the additional components, if larger effects would be obtained in families with more severe problems. However, it may also have led us overestimate the potential effects of the additional components, if smaller effects would be obtained in families with more persistent problems. Consequently, the present outcomes may not generalize to the most severe SP participants and to normal clinical practice. Third, there were differences in financial struggles, negative parenting and time between home visit A and B between the experimental and control group that may have influenced our ability to detect intervention effects, although we controlled for pretest scores in our analyses. Finally, because we had only two conditions, we could not distinguish between the effects of the individual intervention components. Questions about the unique individual effects of the separate components (e.g., whether effects on parenting stress come indeed from the relaxation component, or also from the anger management component) will need to be tested in future research. In addition, it is unclear how the number of and spacing between home visits and the timing of assessment of outcome effects in the present study have influenced the outcomes. Although previous studies have cautiously shown that more sessions is not always better (see e.g. Van der Put et al., 2018), little is known about the optimal time between sessions and the optimal timing of outcome assessment afterwards. Future research should therefore preferably take a mixed methods approach, to include multiple perspectives to explain mixed or unexpected outcomes (or a lack thereof) and provide a better understanding of the facilitators and barriers to implementation that have emerged in the present study.

In sum, the proposed intervention components provide preliminary direction for increasing the effectiveness of home visitation program SP to reduce parenting stress. The long-term added value of the components for reducing child maltreatment remains unclear. Further research is necessary to understand why the additional intervention components may have undermined mothers’ feelings of parental sense of competence and have increased parental anger, and how intervention components targeting parental anger and PTSD symptoms could be changed or expanded to successfully reduce of parental anger and post-traumatic stress symptoms and their impact on the risk for child maltreatment.

## Supplementary Information

Below is the link to the electronic supplementary material.

Supplementary file1 (PDF 309 KB)

## Data Availability

The data that support the findings of this study are available from the last author, CvdP, upon reasonable request.
