# Supplementary material for: Adding Structured Components to Home Visitation to Reduce Mothers’ Risk for Child Maltreatment: a Randomized Controlled Trial
Source: J Fam Violence. 2023 Feb 13:1–14. Online ahead of print. doi: 10.1007/s10896-023-00509-7 (PMC9924864; doi:10.1007/s10896-023-00509-7)
Supplement: Supplementary file 1 — Supplementary file1 (PDF 309 KB) [file 10896_2023_509_MOESM1_ESM.pdf]

Supplementary materials

Adding Structured Components to Home Visitation to Reduce Mothers' Risk for Child Maltreatment: A Randomized Controlled Trial

Journal of Family Violence

Trudy van der Stouwe[1], Patty Leijten[1], Jessica J. Asscher[2], Maja Deković[2], Claudia E. van der Put[1]

[1] Research Institute of Child Development and Education, University of Amsterdam, Post Box 15776, 1001 NG Amsterdam, The Netherlands

[2] Clinical Child and Family Studies, Utrecht University, Post Box 80140, 3508 TC Utrecht, The Netherlands

Corresponding author: Trudy van der Stouwe, t.vanderstouwe@uva.nl.

Appendix A

Participating Nurses, their Reports on Participant Selection Bias and Reasons for Non-Participation of Supportive Parenting Mothers

	n	%
**Participating nurses**	**93**	**100**
Trained in the additional intervention components	63	68
Received online booster training	30	33
**Nurses contacted about non-participation**	**93**	**100**
Nurse provided data about non-participants	53	57
Nurse did not provide data about non-participants	40	43
Reasons:		
Nurse could not be reached	16	40
Nurse did not want to participate anymore	6	15
Nurse had no Supportive Parenting cases during the study	13	33
Nurse felt too busy for participating in the study	2	5
Nurse could not remember number/reasons for non-participants	3	8
**Reported number of non-participating mothers**	**272**	**100**
Nurse did not ask to participate	158	58
Reasons:		
Family situation was deemed too complex/multi-problem	70	44
Language barrier	43	27
Nurse forgot to ask	21	13
Nurse was struggling to obtain basic conditions for intervention	10	6
Additional contents did not match Supportive Parenting contents	9	6
Nurse could not take another strain on her workload	5	3
Mother declined participation	114	42
Reasons:		
Had too much going on in their life	56	67
Said they wanted to participate but did not sign up	29	35
Did not feel like it	18	22
Unknown	9	11

Note.

Appendix B

Mode of Delivery, Topics in Supportive Parenting Home Visits, and Use of Additional Components for the Experimental and Control Group

	EXP		CTRL			
	n	%	*n*	%	χ^2	*p*
**Mode of delivery**						
Home visit A					.01	.924
Home visit	58	82	47	81		
Phone/video call	13	18	11	19		
Home visit B					.92	.336
Home visit	42	69	43	77		
Phone/video call	19	31	13	23		
**Generic topics in 2 home visits**						
Positive feedback	34	46	33	55	1.09	.297
Stress	37	50	25	42	.926	.336
Anger	16	22	8	13	1.55	.213
Trauma	17	23	3	5	8.43	.004**
**Added intervention components**						
Parental sense of competence						
In 2 home visits	20	27	6	10	6.14	.013*
In at least 1 home visit	48	65	20	33	13.18	.000***
Perceived stress						
In 2 home visits	24	32	3	5	15.50	.000***
In at least 1 home visit	52	70	12	20	33.56	.000***
Mother used the exercise	23	31	5	8	10.37	.001**
Parental anger						
In 2 home visits	9	12	1	2	5.29	.022*
In at least 1 home visit	29	39	14	23	3.82	.051
PTSD symptoms						
In 2 home visits	16	22	5	3	4.43	.035*
In at least 1 home visit	51	69	12	20	31.83	.000***
When PTSD checklist was used$^+$						
Score above threshold	29	56	10	83	2.89	.089
When mother scored above threshold						
Nurse thinks > 75% chance of seeking help$^\wedge$	23	79	9	90	.58	.448
All 4 components						
In 2 home visits	2	3	0	0	1.65	.199
In at least 1 home visit	16	22	2	3	9.53	.002**

Note. EXP = Experimental group. CTRL = Control group. HA = Home visit A. HB = Home visit B.
* $p < .05$. ** $p < .01$. *** $p < .001$.
$^+$ = EXP $n = 51$, CTRL $n = 12$.
$^\wedge$ = EXP $n = 29$, CTRL $n = 10$.

Appendix C

Number of Days between Assessment and Home Visit Points and Time since T1 for the Experimental and Control Group

	EXP			CTRL				
	n	*M*	*SD*	*n*	*M*	*SD*	*t*	*p*
Between…								
T1-HA	65	18.78	20.08	68	17.26	20.64	.42	.679
HA-T2	69	20.10	8.11	52	18.87	10.99	.71	.478
T2-T3	45	62.91	51.73	33	38.18	35.59	2.36	.021*
T3-HB	44	15.14	14.29	33	15.48	14.90	-.10	.917
HB-T4	57	28.04	25.38	52	29.52	35.27	-.25	.800
Since T1 and…								
HA	65	18.78	20.08	68	17.26	20.64	.42	.679
T2	64	38.52	20.77	54	36.24	19.54	.61	.544
T3	41	89.10	49.39	34	68.97	39.04	1.93	.058
HB	54	93.72	47.97	53	75.45	32.84	2.29	.024*
T4	67	147.78	86.30	58	115.09	57.48	2.52	.013*

Note. EXP = Experimental group. CTRL = Control group. HA = Home visit A. HB = Home visit B.
* $p < .05$. ** $p < .01$. *** $p < .001$

Appendix D

Figures 2a-2d

Primary Outcomes over Time per Group

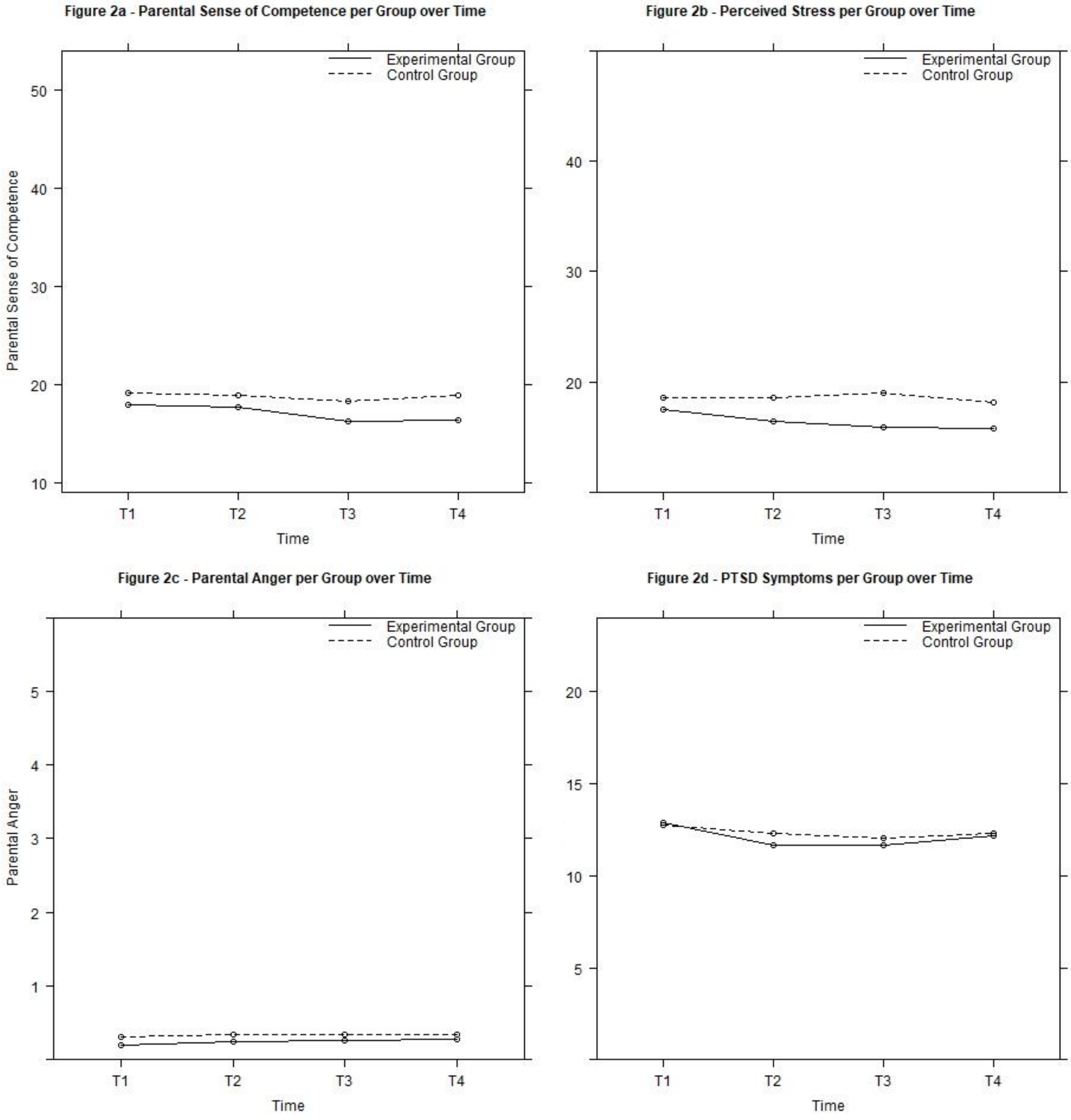

Note. Y-axis min-max = Scale min-max.

Figures 3a-3c

Secondary Outcomes over Time per Group

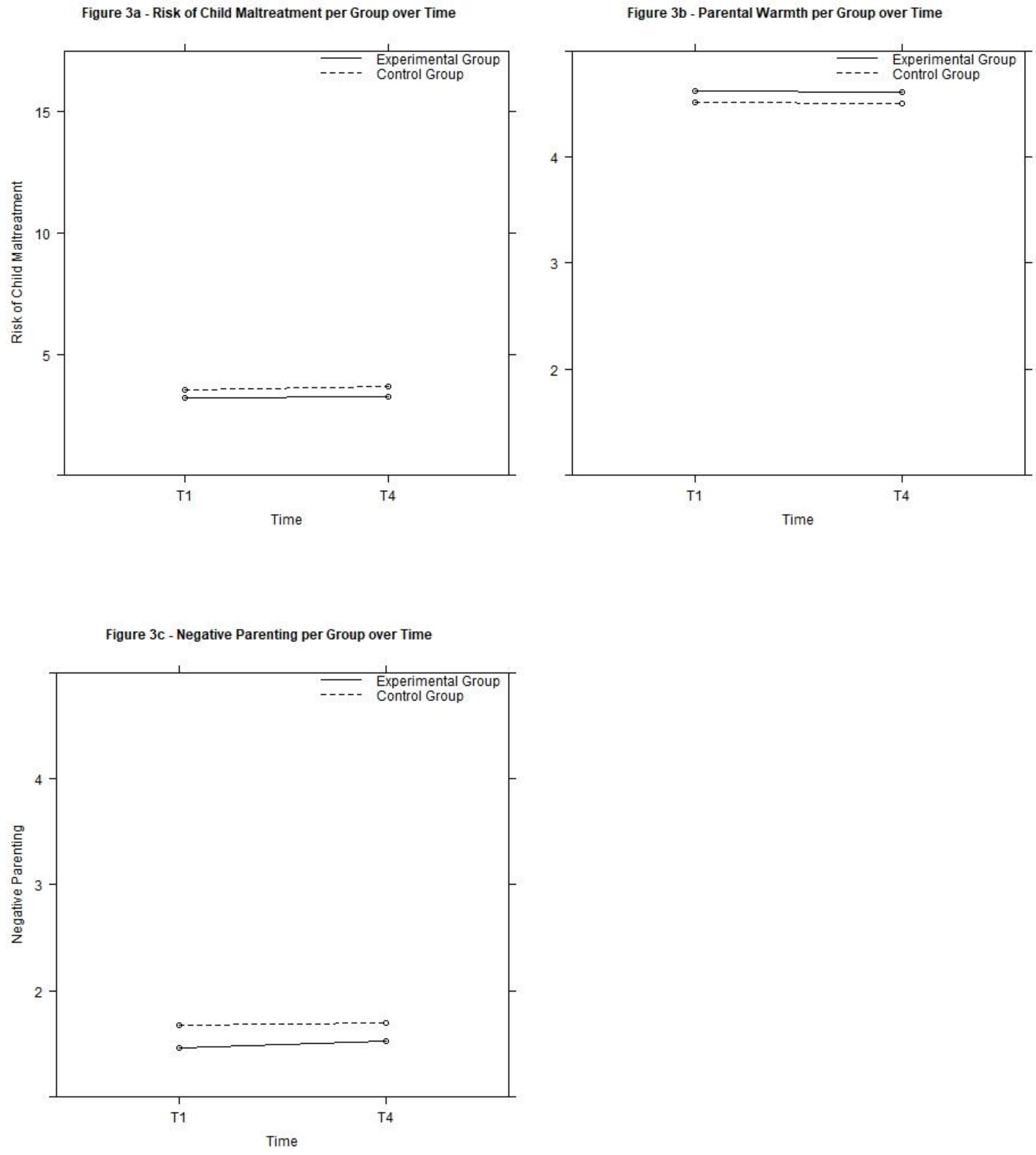

Note. Y-axis min-max = Scale min-max.
